# Relationship between energy availability, energy conservation and cognitive restraint with performance measures in male endurance athletes

**DOI:** 10.1186/s12970-021-00419-3

**Published:** 2021-03-18

**Authors:** Iva Jurov, Nicola Keay, Vedran Hadžić, Darjan Spudić, Samo Rauter

**Affiliations:** 1 Faculty of Sport, University of Ljubljana, Gortanova 22, 1000 Ljubljana, Slovenia; 2 Department of Sport and Exercise Sciences, Durham University, Durham, UK

**Keywords:** Energy availability, Performance, Endurance athletes, Relative energy deficiency, Cognitive restriction

## Abstract

**Background:**

Low energy availability in male athletes has gained a lot of attention in recent years, but direct evidence of its effects on health and performance is lacking. The aim of this research was to objectively measure energy availability (EA) in healthy male endurance athletes without pre-existing relative energy deficiency signs during pre-race season.

**Methods:**

Twelve trained endurance athletes (performance level 3, 4, and 5) participated in the cross-sectional controlled laboratory study. Fat-free mass, exercise energy expenditure, and energy intake were measured to calculate EA. Resting energy expenditure was measured and estimated to assess energy conservation. Three specific performance tests were used to assess endurance, agility, and explosive strength performance. For psychological evaluation, the Three Factor Eating Questionnaire and a short Well-being questionnaire were completed.

**Results:**

Mean EA was 29.5 kcal/kg FFM/day. The majority (66.6%) had EA under the threshold for low EA in females. Critical cognitive restraint (≥13) was reported by 75% of participants. There were no differences in performance, blood values, or psychological evaluation when subjects were divided into two groups divided by EA = 30 kcal/kg FFM/day. Cognitive restraint was negatively associated with measured resting energy expenditure and energy conservation (*r* = −.578, *p* = .025 and *r* = −.549, *p* = .032, respectively).

**Conclusions:**

The mean EA measured in this study supports the theory that the threshold for low EA in endurance male athletes might be under the threshold for females. In addition, we confirmed cognitive restraint could be useful for early detection of energy conservation. The high cognitive restraint as measured in our sample stressed the need of eating behavior screening in endurance athletes in order to reduce risk of any disordered eating patterns.

**Supplementary Information:**

The online version contains supplementary material available at 10.1186/s12970-021-00419-3.

## Background

Endurance athletes are at risk for development of a syndrome called *relative energy deficiency in sport* (RED-S) [1]. *Low energy availability* (LEA) is the underlying cause for RED-S [2]. Estimated prevalence of LEA is high [3] but methodology for assessment is not universal and often based on questionnaires and subjective estimation. The threshold for LEA is known in female athletes (30 kcal/kg fat-free mass(FFM)/day) [4], but the equivalent in men is yet to be confirmed. While diagnosing athletes with obvious RED-S signs and symptoms is relatively straightforward, detecting LEA before detrimental health issues arise presents a greater challenge. It is also unclear how and when LEA affects performance. Clearly, performance is of the greatest interest to athletes and their coaches. Unfortunately, there is currently little research directly observing LEA’s association with performance. Our current knowledge on performance effects is mostly theoretical [2, 5]. Objective methodology for measuring EA is the only way to discover the threshold of LEA in men. After a threshold (or a range for LEA) is confirmed, we will then be in a better position to elucidate the effects on performance more readily. There is speculation that performance effects could arise before clinical signs of poor well-being. This is why measuring EA status in apparently healthy athletes could provide insight into the association of EA with performance.

In times of energy deprivation, energy is spared at the cost of growth and reproduction [6]. Metabolic changes in the body can result in energy conservation in order to ensure homeostasis. There are more mechanisms underlying energy conservation [7], which is detected by ratio of measured resting energy expenditure (mREE) and predicted resting energy expenditure (pREE) – the mREE/pREE ratio. It was previously reported that energy conservation could be a useful marker for detecting LEA. In female athletes mREE/pREE ratio < 0.9 was associated with relative energy deficiency [8, 9] and with poor aerobic performance in competitive female cyclists [10], but there were no reported cutoff values in men.

The primary endpoint of our study was to objectively measure energy availability (EA) in trained male endurance athletes without pre-existing RED-S signs during pre-race season and to evaluate and quantify possible relationships between measured EA and mREE/pREE, specific blood marker and performance parameters. Our secondary endpoint was analysis of the relationship between cognitive restraint and EA, as there is evidence in the literature suggesting that psychological questionnaires might be a better tool for RED-S screening than endocrine markers [11].

## Methods

### Study design

**Fig. 1:**
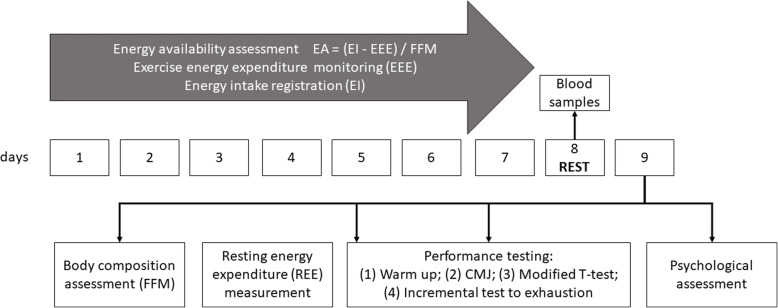
The timeline of all procedures and measurements

This was a cross-sectional controlled laboratory study. With unchanged living and training conditions, subjects reported energy intake (EI) by completing dietary diaries for 7 consecutive days [12] (Fig. 1). During this period, exercise energy expenditure (EEE) was monitored during all training units. After 7 days, blood samples were drawn and after 1 day of rest (on day 9), body composition was assessed and REE was measured, followed by three performance tests for determining basal performance. At the end of the study, participants completed psychological questionnaires.

### Participants

**Fig. 2:**
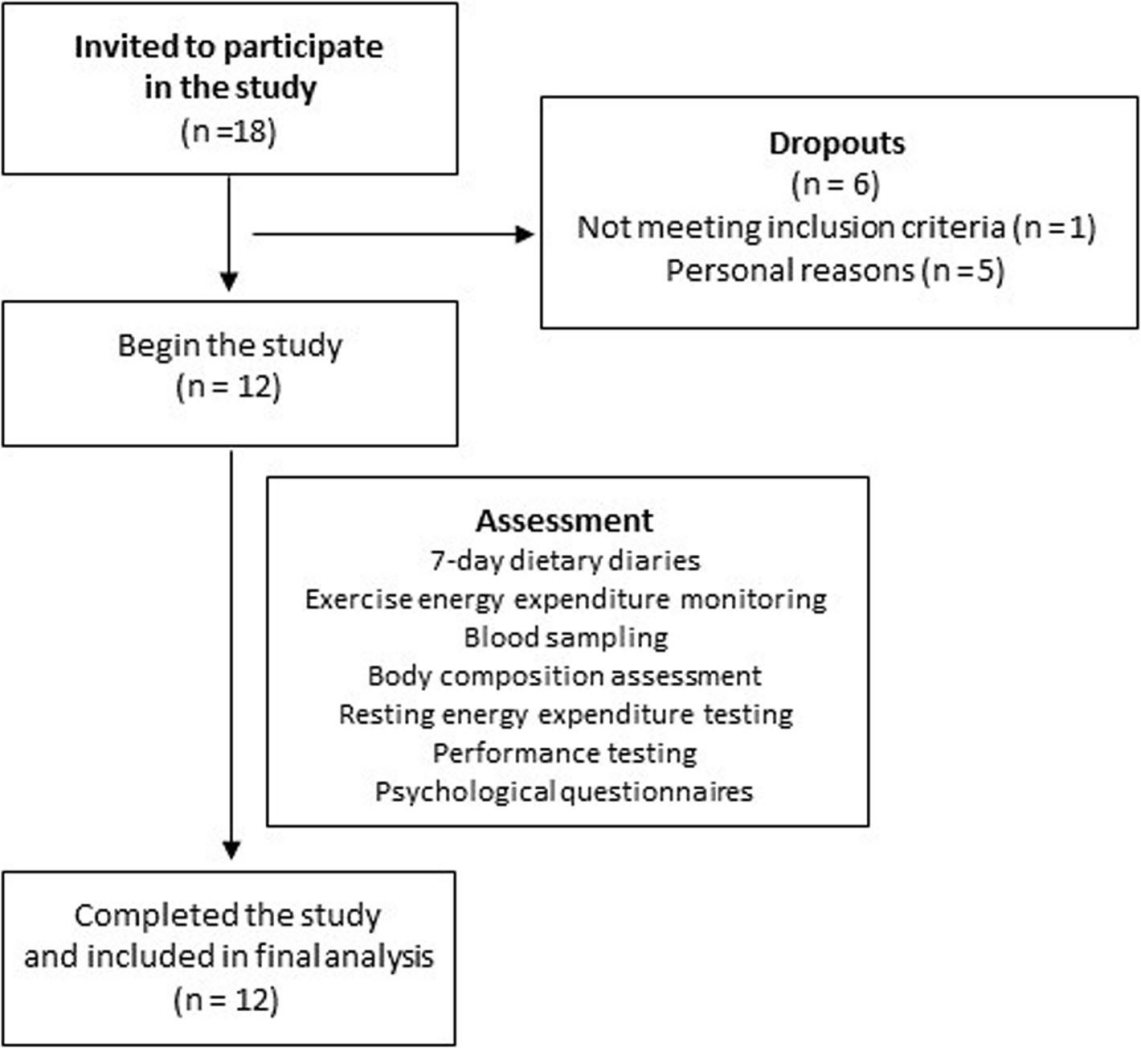
Flowchart of participant enrollment in the study

Eighteen (*N* = 18) males were invited to participate in this research. Inclusion criteria for participation in the study are presented in Table 1 and flowchart of enrollment in Fig. 2.

**Table 1:** Inclusion criteria for participants

Sex	Male
Age	18–35 years
Performance level	Well trained; with VO2max 55–64.9 ml/kg/min; performance level 3 or more [13]
BMI	1. BMI 19–25 kg/m^2^; in normal range for adult males)
Body Fat Percentage	2. 5–20%
Health status	1. No acute disease or chronic disease in relapse (allowing only for chronic diseases that are stable and not affecting performance) 2. At the time of procedures be free of injuries and no injuries in previous three months that could affect performance
Additional criteria	3. Stable body mass for the last 12 months
	4. Not undertaking any specific diet regime
	5. At the time of procedures will refrain from alcohol consumption and any drug or other substance use
	6. Complete all procedures and report any factors that could influence changes in blood values or performance (lack of motivation due to psychological factors, factors in between measurements that could influence results etc.)

All participants needed to sign an informed consent before commencing all protocols for allowing data to be gathered and analyzed anonymously. This research complied with the declaration of Helsinki. National medical ethical approval was acquired before the start of the study (No. 0120–202/2020/5).

### Subject involvement

Subjects were invited to participate in the study through national cycling and triathlon organizations, professional cycling team’s coaches. The information was also disseminated through faculty’s laboratory, where national best endurance athletes regularly perform various testings.

Subjects were informed of all procedures and were selected based on inclusion criteria, high motivation and compliance.

### Procedures

#### Energy availability calculation

All procedures were carried during a 9-day period (Fig. 1). Participant EI was measured by completing dietary diaries for 7 consecutive days [12]. All participants received detailed information on how to complete the diary and how to weigh food or measure its quantity with the help of cups and other measuring tools. They were asked to provide photographic evidence of all food and liquid ingested in that time. EI data was analyzed with Foodworks 9 Professional Edition (version 9.0.3973, Xyrix Software, Australia). During this same period EEE was estimated from heart rate using wearable heart rate monitors during all exercise sessions (Polar V800, Polar Electro, Kempele, Finland). EA was calculated as EA = (EI-EEE)/FFM.

#### Performance testing

To test performance, three different tests were chosen to assess explosive power of lower extremity (Countermovement jump), motor task execution time (agility t-test) and maximal aerobic capacity (incremental aerobic endurance test). The details of warm-up protocol and tests can be found in the Additional file 1.

First, CMJ test was performed using a bilateral force plate system (Type 9260AA, Kistler Instrumente AG, Winterthur, Switzerland) with Kistler MARS software (S2P Ltd., Ljubljana, Slovenia) to acquire ground reaction force*.* Each subject has performed three to five maximal counter movement jumps before the testing.

Second, to asses motor task execution time, validated modified agility t-test was used, as described by Haj-Sassi, et al. (2011). The time of best repetition (seconds) were used in further analysis.

After 1 h of rest, endurance was measured with the incremental test to exhaustion. Heart rate, ventilatory, and gas data were collected during the incremental test with metabolic cart (V2 mask (Hans Rudolph, USA), K5 (Cosmed, Albano Laziale, Rome, Italy) with Quark 8.1. PC software support) on a cycle ergometer (Cyclus 2, Leipzig, Germany).

#### Blood samples

On day 8, venous blood samples were drawn in the morning at 9 am in a fasted state to assess complete blood count, ferritin, serum iron (Fe), triiodothyronine (T3), thyroid stimulating hormone (TSH), morning testosterone, fasting insulin, insulin like growth factor 1 (IGF-1) and 9 am cortisol. Blood was collected using standard clinical procedures. Haemoglobin was analysed with Sysmex XN-550 (photometric detection, EDTA tubes), iron with Cobas c501 (colorimetric analysis, serum tubes), ZSH, T3, testosterone, cortisol and ferritin with Cobas e411 (electrochemiluminescence immunoassay, serum tubes). Serum insulin level was analyzed with a double antibody RIA (serum tubes) and for IGF-1 the RIA kit (serum tubes) was used.

#### Body composition assessment

Body composition was assessed using tetra polar eight point tactile bioelectrical impedance device InBody 720 (Biospace, Seul, South Korea) on day 9. Prior to body composition measurement, participants received instructions how to be adequately hydrated to enable precise measurement of FFM and body fat percentage that were used in further analysis.

#### Resting energy expenditure assessment

REE was measured with indirect calorimetry (V2 mask (Hans Rudolph, USA), K5 (Cosmed, Albano Laziale, Rome, Italy) with Quark 8.1. PC software support) based on the Weir equation [14, 15]. The measurement was performed in a thermoneutral environment, in silence, between 6.00 and 9.00 a.m., after 12 h of fasting [16]. It lasted 30 min and the final 20 min were used for REE measurement [17]. During REE measurement, respiratory quotient was monitored since measures under 0.70 or above 1 suggest protocol violations or inaccurate gas measurement [17]. To obtain predicted REE (pREE), a Harris-Benedict equation was used [18]. The mREE/pREE ratio was then calculated for further analysis.

#### Psychological assessment

The Three Factor Eating Questionnaire (TFEQ-R18) and Well-being questionnaire were used for psychological assessment [19, 20]. TFEQ-R18 was used to detect early changes in eating behaviors and has three subscales including cognitive restraint, disinhibition and susceptibility to hunger, with higher scores indicating greater eating disturbances in participants. The subscale of interest was cognitive restraint. General well-being was assessed by a simple questionnaire as recommended by Hooper and Mackinnon (1995) including six subjective ratings (fatigue, sleep, stress, muscle soreness, mood and morning erections) on a 1–5 scale. The last item about morning erections was added to the original set as proposed by a study on professional rugby players [21] (Additional file 2).

### Data analysis

All data were analyzed using the IBM SPSS Software for Windows (version 21, SPSS Inc., Armonk, New York, USA). Categorical variables are displayed as numbers and percentages, and numeric variables are presented as means and standard deviations. All numeric variables were first checked for normality of distribution with Shapiro-Wilk’s test. Pearson’s correlation coefficient was computed to assess the relationship between EA and obtained performance, laboratory, body composition and psychological parameters. Based on the EA value, the subjects were later divided into two subgroups (with EA ≥ 30 kcal/kg FFM/day and with EA < 30 kcal/kg FFM/day). The possible differences in performance, blood, anthropometric, body composition, and psychological parameters between those two groups were analyzed using the t-test for independent samples. The significance level was set at *p*-values < 0.05 for all calculations.

## Results

The means and standard deviations of all obtained parameters are presented in Table 2.

**Table 2:** All obtained parameters in the study design

	Parameters	Mean	Std. Dev.
**Anthropometrics and body composition parameters**	Age (years)	27.5	5.7
	Body height (cm)	179.8	4.4
	Body mass (kg)	71.8	3.6
	Fat-free mass - FFM (kg)	64.5	3.7
	%FFM (%)	89.8%	2.5%
	Percentage body fat (%)	10.2%	2.5%
**Energy and metabolic parameters**	Energy intake (kcal/day)	3078	520
	Exercise energy expenditure (kcal/day)	1173	420
	mREE (kcal/day)	1824	357
	pREE (kcal/day)	1770	68
	mREE/pREE ratio	1.03	0.21
	Energy availability (kcal/day/kg FFM)	29.5	7.9
**Blood samples**	Haemoglobin (g/L)	146.83	9.28
	S-Iron (μmol/L)	22.91	3.93
	S-TSH (mIU/L)	2.37	0.67
	S-T3 (pmol/L)	4.46	0.54
	S-Testosterone (nmol/L)	17.74	3.53
	S-Cortisol (nmol/L)	454.37	88.46
	S-Feritin (μg/L)	129.43	99.03
	Insulin (mE/L)	2.84	1.33
	IGF-1 (μg/L)	186.17	55.21
	IGF-1 SD	−0.19	0.81
**Performance parameters**	VO_2max_ (ml/min/kg)	67.49	6.74
	PO (W)	402.50	40.03
	RPO (W/kg)	5.60	0.47
	AT (ml/min/kg)	47.10	5.99
	RC (ml/min/kg)	57.48	7.12
	[La]_max_ (mmol/l)	10.80	2.46
	[La]_5min_ (mmol/l)	11.29	2.07
	Modified t-test (seconds)	6.49	0.40
	Countermovement jump height (cm)	32	5
**Psychological assessment**	Well-being score	17.83	3.54
	TFEQ-18 score	42.58	7.13
	TFEQ-18 cognitive restraint subscale	14.75	4.18

*mREE* measured resting energy expenditure, *pREE* predicted resting energy expenditure, *VO*_*2max*_ maximal oxygen consumption, *PO* peak power output, *RPO* relative power output, *AT* anaerobic threshold, *RC* respiratory compensation point, *[La]*_*max*_ lactate concentration at the end of the test, *[La]*_*5min*_ lactate concentration 5 min after the end of the test, *TFEQ* the three factor eating questionnaire

**Fig. 3:**
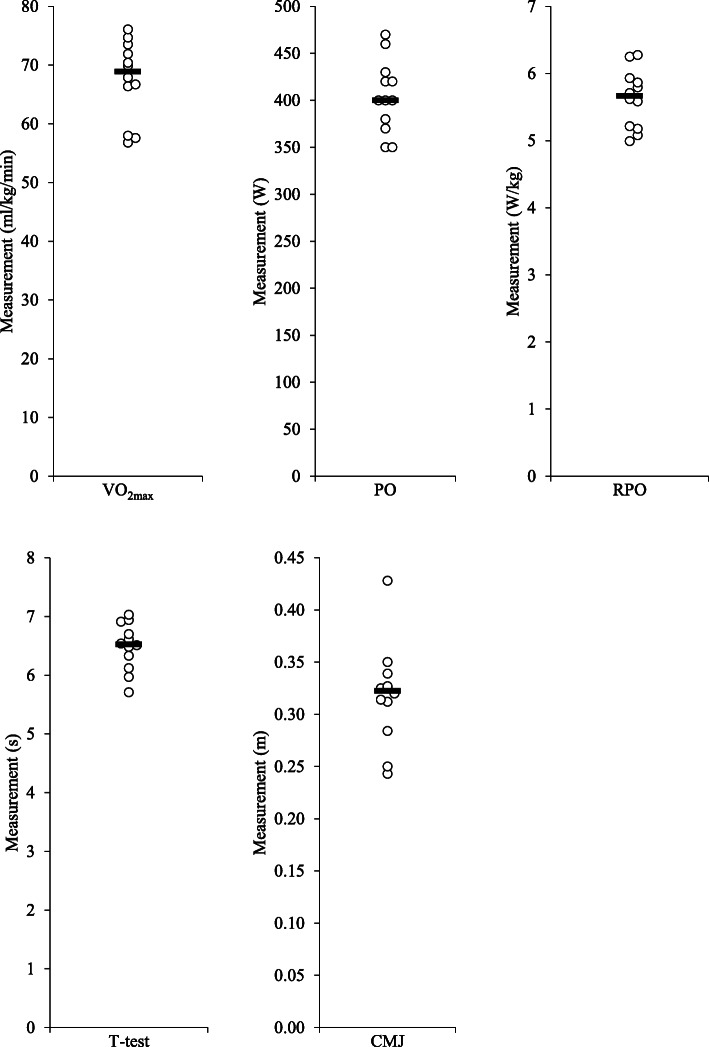
Scatterplots of performance parameters (PO - peak power output, RPO - relative power output, VO_2max_ – maximal oxygen uptake, CMJ – countermovement jump)

Our results indicate that this was a sample of well-trained healthy endurance athletes. Average training time was 2 h and 4 min (80.6% spent cycling, 9.3% running and 10.1% swimming). Furthermore, mean VO_2max_ showed that 25% of participants are at the performance level 3 (VO_2max_ between 55.0 and 64.9 ml/min/kg), 33.3% at the performance level 4 (VO_2max_ between 65 and 71 ml/min/kg) and 41.6% are professional athletes with performance level 5 (VO_2max_ > 71 ml/min/kg) (Fig. 3). In addition to endurance performance, we report good jumping capacity as well as the agility with motor task execution times within the normal range expected for the sex and age of the participants (mean time 6.49 s). Hormone levels were within the normal range without any pathological findings, with only one participant with testosterone levels in the lower quartile reference range. Serum iron levels were also in the healthy range, and there were no pathological findings in the complete blood count (not presented in Table 2).

Our main findings are related to energy and metabolic parameters in this healthy, well-trained sample of endurance athletes. EI was 3078 kcal and EEE was 1173 kcal. Calculated energy availability was 29.5 kcal kg FFM (95% CI 25.6 to 33.4).

Pearson correlation analysis did not show any significant correlations between anthropometric parameters, performance parameters, hormone levels, or any other blood parameter and EA. However, we found that EA has significant negative correlation with EEE (*r* = −.618, *p* = .016) and that EI had significant positive correlation with cognitive restraint subscale of TFEQ (*r* = .559, *p* = .03), while it was negatively correlated with mREE (*r* = −.578, *p* = .025) and mREE/pREE ratio (*r* = − .549, *p* = .032). Nine (*n* = 9; 75%) participants reported critical cognitive restraint, which is any value ≥13 indicating possible LEA presence.

A t-test for independent samples was used to compare subgroups of subjects with EA ≥ 30 kcal/kg FFM/day (*n* = 6) and subjects with EA < 30 kcal/kg FFM/day (*n* = 6). There were no significant differences in any of the compared parameters.

## Discussion

This study design was set to measure actual EA in healthy endurance athletes in the pre-race period. The mean EA measured in this study supports the theory that the threshold for LEA in male endurance athletes might be below the threshold set for females. In addition, we confirmed cognitive restraint could be useful for early detection of energy conservation. The high cognitive restraint as measured in our sample stresses the need of eating behavior screening in endurance athletes in order to reduce the risk of disordered eating patterns and eating disorders.

### Energy markers

As expected, anthropometric measurements showed the subjects were lean (body fat M = 10.2% ± 2.5%). They trained more than 2 h per day, expending 1173 kcal on daily average. According to physiological parameters obtained at the incremental test, subjects were trained (*n* = 3), well-trained (*n* = 4) and professional athletes (*n* = 5) [13]. Their mean EA was low - it was 29.5 kcal/kg FFM/day and according to Melin et al. [22] it would be considered clinically relevant and symptomatic. Two thirds of the subjects (66.6%) had EA < 30, which is in accordance with studies showing prevalence of LEA is high in athletes [3]. However, Fagerberg (2018) has suggested that in male athletes a prolonged EA < 25 kcal/kg FFM/day could be the critical value, although this paper was referring to strength and not endurance athletes. Our findings support the Fagerberg proposal, but the true cut-off value of EA will have to be investigated more thoroughly in future studies, so that these predictions are supported by relevant evidence. Greater EEE was associated with lower EA (*r* = − 618, *p* = .016). This situation raises concern because pre-race season EEE should be coupled with sufficient EI to ensure optimization of adaptation to training.

We also aimed to find any association between energy conservation and EA. In females, LEA and mREE/pREE are associated and this association has been shown primarily on women with amenorrhoea [9, 23–27]. In males, it is not yet clear if EA and energy conservation are indeed connected. Furthermore, it remains an open question whether the cut-off point of mREE/pREE < 0.9 could be used as a potential screening marker for LEA. In our sample, mean mREE/pREE was 1.03, which was unexpected when considering mean EA was under proposed optimal EA value (≥40 kcal/kg FFM/day) for men [22]. In this study, we suggest there are two possible reasons why we failed to show this association. The first is that while mREE/pREE might indeed be correlated to LEA, we do not know the threshold for LEA in men. From our study we suspect the mean EA values were simply not low enough for the marker mREE/pREE to show energy conservation (i.e. mREE/pREE < 0.9 or even lower [24]). The second reason could be that greater sample size might reveal more since there was only one subject with mREE/pREE< 0.9 in our sample. However, since our participants were healthy athletes without any LEA signs, the likely explanation is that they did not have LEA. Therefore, the mean mREE/pREE was close to 1, which is an expected value when there is no energy conservation. Although the athletes had a mean EA =30 kcal/kg FFM/day, this suggests that LEA was not present.

### Cognitive restraint

Cognitive restraint means that a person consumes less than they like to and does not tell anything about energy balance [28]. Gibbs et al. [25] showed that cognitive restraint ≥13 might be a useful marker for LEA in exercising women. We were not able to show an association between cognitive restraint ≥13 and lower EA in our sample of male endurance athletes. This might be due to high mean cognitive restraint in our sample. 75% of our participants had greater values than 13, which is worrying since this is their eating behavior in the pre-race season preparation period when the pressure on optimal body mass is not yet high. The lowest body mass should be achieved in the competitive season. Secondly, subjects with greater EI had higher cognitive restraint (*r* = .599, *p* = .03). Expressing high cognitive restraint could lead to disordered eating. It is known that when entering pre-race season preparation period, endurance athletes have high energy demands due to high training volume. This should be coupled with satisfactory EI in order to achieve good training adaptation and recovery. High cognitive restraint in our sample suggests athletes might intentionally restrict their EI in order to achieve optimal body composition. Finally, the bigger cognitive restraint, the lower were mREE and mREE/pREE (*r* = −.578, *p* = .025 and *r* = −.549, *p* = .032, respectively). cognitive restraint could thus be associated with energy conservation as measured by mREE and expressed by mREE/pREE.

### Performance

There were no correlations between any performance variables and EA or energy conservation. We suspect that a larger sample size might reveal more. Since it is very difficult to find athletes who meet all the inclusion criteria and are willing to undergo all the procedures of an objective EA measurement, reducing EA in the same individuals might be an easier way to see if performance is related to EA or energy conservation. This could also be useful to see if different performance modalities respond differently to EA changes.

### What is the threshold for LEA?

In order to detect a possible threshold, we divided subjects into two groups separated by EA = 30 kcal/kg FFM/day. We failed to find any differences in endurance, strength, agility performance or blood parameters. In addition, there were no differences in testosterone’s functional health effects (morning erections). This indicates that EA = 30 kcal/kg FFM/day might not be the actual threshold for LEA in men as proposed before by Viner et al. [19]. A lower threshold might be more accurate. Unfortunately, dividing subjects based on a threshold of 15 kcal/kg FFM/day as suggested by Koehler et al. [29] was not possible for analysis since only one of the subjects reached EA < 15 kcal/kg FFM/day. The possibility that the threshold in men could be lower is also supported by blood test results. All measured parameters (full blood count, ferritin, serum Fe, T3, TSH, morning testosterone, fasting insulin, IGF-1, 9 am cortisol) were normal in all participants, despite mean EA was lower than 30 kcal/kg FFM/day. LEA must persist over a long period of time to produce health or performance changes, and it is not possible to draw definitive conclusions from a single EA objective measurement. In further research, such measurements should be repeated more often in the same participants to see if their mean EA is indeed lower than 30 kcal/kg FFM/day. Also, future research should focus on inducing lower EA in the same participants. This could also be the next step in order to find if a lower threshold indeed shows differences in hormones and/or performance.

### Limitatons

We would like to acknowledge that a more homogenous sample of only one endurance discipline would be more optimal. As known in this research area, finding participants that are willing to accept the high burden of EA measurement is challenging. Still, this is one of the largest sample sizes of trained endurance athletes when measured with objective EA methodology. This is why we believe this paper is an important contribution to current knowledge. In addition, other individual characteristics of athletes influence performance. A study comparing different EA in same individuals would be a more appropriate way to compare performance.

## Conclusions

This paper suggests that psychological evaluation such as cognitive restraint in the TFEQ might be more appropriate where there are no signs of RED-S for assessing energy conservation and possible suboptimal EA. This study adds to our knowledge that the threshold for LEA in men is indeed probably lower than 30 kcal/kg FFM/day. Further studies with objective measurement of EA in athletes with apparent LEA or laboratory induced LEA should be performed to determine the threshold for LEA or refute its existence. Practical application of this study is that coaches and their athletes might benefit from cognitive restraint assessment in order to prevent energy conservation.

## Supplementary Information

**Additional file 1.**

**Additional file 2.**

## Data Availability

Data are available upon reasonable request.

## Abbreviations

[La]_5min_: Lactate concentration 5 minutes after the end of the test
[La]_max_: Lactate concentration at the end of the test
AT: Anaerobic threshold
CMJ: Countermovement jump
CR: Cognitive restraint
EA: Energy availability
EEE: Exercise energy expenditure
EI: Energy intake
Fe: Serum iron
FFM: Fat-free mass
IGF-1: Insulin like growth factor 1
LEA: Low Energy Availability
mREE: measured resting energy expenditure
PO: Peak power output
pREE: predicted resting energy expenditure
RC: Respiratory compensation point
RED-S: Relative Energy Deficiency in Sport syndrome
RPO: Relative power output
T3: Triiodothyronine
TFEQ-R18: Three Factor Eating Questionnaire
TSH: Thyroid stimulating hormone
VO_2max_: Maximal oxygen uptake
