# Supplementary material for: Relationship between energy availability, energy conservation and cognitive restraint with performance measures in male endurance athletes
Source: J Int Soc Sports Nutr. 2021 Mar 18;18:24. doi: 10.1186/s12970-021-00419-3 (PMC7977281; doi:10.1186/s12970-021-00419-3)
Supplement: Supplementary file 1 — Additional file 1. [file 12970_2021_419_MOESM1_ESM.docx]

**Additional file 1**

Performance tests in detail

To test performance, three different tests were chosen to assess vertical jump height (explosive power of lower extremities), motor task execution time (agility) and maximal aerobic capacity (aerobic endurance). The details of warm-up protocol, countermovement jump (CMJ), agility t-test and the incremental test can be found in the supplementary file.

Performance tests were performed in this exact order for each participant following the general warm up consisting of 2 min of cycling on a stationary bike at a 50 revolutions per minute at approximately 1.5 W/kg. Then they performed arm, hip, knee and ankle mobility exercises (10 reps each); dynamic stretches of hip flexors, knee extensors, knee flexors and ankle extensors (10 reps each); and heel raise, squat, crunch resistance exercises (10 reps each).

Countermovement jump (CMJ) test was performed using a bilateral force plate system (Type 9260AA, Kistler Instrumente AG, Winterthur, Switzerland) with Kistler MARS software (S2P Ltd., Ljubljana, Slovenia) to acquire ground reaction force*.* Each subject performed three to five maximal counter movement jumps before the testing. For CMJ data were sampled at 1000 Hz, filtered using a moving average filter with 50-ms window and analyzed using the built-in module for CMJ. Test execution was supervised from the experienced researcher to improve proficiency in jumping technique (1). Before each jump, participants were instructed to stand up straight and still on the center of the force plate with their hands akimbo. This hand position remained the same during the entire movement. From this position, participants initiated a fast downward movement until a crouching position with a knee angle of about 90°, followed by a jump for maximal height as quickly and explosively as possible. Three valid trials were performed with one-minute recovery period. The main outcome measure was CMJ height in centimeters that was calculated from the maximum velocity (2).

To asses motor task execution time, validated modified agility t-test was used, as described by Haj-Sassi, et al. (2011). The T-test was performed after 5 minutes rest from vertical jump testing without additional warm up. Each participant had three trials (each trail consisting of two sprints separated by 30 seconds rest). The rest between trials was 90 seconds. Cones were placed in a T-shape layout, 5 and 2.5 m apart, respectively. Subjects were instructed to sprint and change direction as fast as possible. They began with both feet 0.3 m behind the starting line (A). At their own discretion subject sprinted forward to cone B and touched the base of it with the right hand. Facing forward and without crossing feet, they shuffled to the left to cone C and touched its base with the left hand. Subjects then shuffled to the right to cone D and touched its base with the right hand. They shuffled back to the left to cone B and touched its base. Finally, subjects ran backward as quickly as possible to cross the finish line (cone A) ending the first sprint. The second sprint started after 30 seconds rest. Three such trials separated by 90 seconds rest were performed, all with verbal encouragement. The subject who crossed one foot in to each direction (firstly starting with shuffling to the left) front of the other, failed to touch the base of the cone or failed to face forward throughout, had to repeat the test. The time to complete each repetition was measured using one pair of the electronic timing system sensors (Witty Timing System, Microgate, Bolzano) mounted on tripods. They were set approximately 0.75 m above the floor positioned 2 m apart facing each other on either side of the starting line (A). The time of best repetition (seconds) and three repetition average were used in further analysis.

After 1 hour of rest, endurance was measured with the incremental test to exhaustion. Heart rate, ventilatory, and gas data were collected during the incremental test with metabolic cart (K5, Cosmed, Italy). All measurements were performed in the physiological laboratory with ambient temperature of 21°C. For measuring VO_2max_, the following procedure was performed. After a 15-minute warm-up on bicycle set up on a cycle ergometer (Cyclus 2, Leipzig, Germany), workload constantly increased until volitional exhaustion (100 W + 20 W every minute). Lactate was analyzed in capillary blood drop from the earlobe. Samples were obtained at rest before any physical activities, at the end of the test and 5 minutes after the test. Lactate was analyzed with the blood lactate analyzer Biosen C_Line (EKF Diagnostics, Germany).

1. Mandic R, Jakovljevic S, Jaric S. Effects of countermovement depth on kinematic and kinetic patterns of maximum vertical jumps. J Electromyogr Kinesiol [Internet]. 2015 Apr 1 [cited 2020 Aug 20];25(2):265–72. Available from: /pmc/articles/PMC4355311/?report=abstract

2. García-Ramos A, Štirn I, Padial P, Argüelles-Cienfuegos J, De la Fuente B, Strojnik V, et al. Predicting vertical jump height from bar velocity. J Sport Sci Med. 2015;14(2):256–62.

3. Haj-Sassi R, Dardouri W, Gharbi Z, Chaouachi A, Mansour H, Rabhi A, et al. Reliability and Validity of a New Repeated Agility Test as a Measure of Anaerobic and Explosive Power. J Strength Cond Res [Internet]. 2011 Feb [cited 2020 Jun 15];25(2):472–80. Available from: http://journals.lww.com/00124278-201102000-00026
