# Supplementary material for: Relationship between energy availability, energy conservation and cognitive restraint with performance measures in male endurance athletes
Source: J Int Soc Sports Nutr. 2021 Mar 18;18:24. doi: 10.1186/s12970-021-00419-3 (PMC7977281; doi:10.1186/s12970-021-00419-3)
Supplement: Supplementary file 2 — Additional file 2. [file 12970_2021_419_MOESM2_ESM.docx]

**Additional file 2**

Well-being subjective questionnaire with scoring system

|  | 5 | 4 | 3 | 2 | 1 |
| --- | --- | --- | --- | --- | --- |
| FATIGUE | very fresh | fresh | normal | more tired than normal | always tired |
| SLEEP QUALITY | very restful | good | difficulty falling asleep | restless sleep | insomnia |
| GENERAL MUSCLE SORENESS | feeling great | feeling good | normal | increase in soreness/tightness | very sore |
| STRESS LEVELS | very relaxed | relaxed | normal | feeling stressed | highly stressed |
| MOOD | very positive mood | a generally good mood | less interested in others &/or activities than usual | snappiness at teammates, family and co-workers | highly annoyed/ irritable/ down |
| MORNING ERECTIONS | >5 per week |  |  | <2 per week | almost never/not present |
